# Quorum sensing activity of *Citrobacter amalonaticus* L8A, a bacterium isolated from dental plaque

**DOI:** 10.1038/srep20702

**Published:** 2016-02-10

**Authors:** Share-Yuan Goh, Saad Ahmed Khan, Kok Keng Tee, Noor Hayaty Abu Kasim, Wai-Fong Yin, Kok-Gan Chan

**Affiliations:** 1 Division of Genetics and Molecular Biology, Institute of Biological Sciences, Faculty of Science, University of Malaya, 50603 Kuala Lumpur, Malaysia; 2 Department of Restorative Dentistry, Faculty of Dentistry, University of Malaya, 50603 Kuala Lumpur, Malaysia; 3 Department of Medicine, Faculty of Medicine, University of Malaya, 50603 Kuala Lumpur, Malaysia

## Abstract

Cell-cell communication is also known as quorum sensing (QS) that happens in the bacterial cells with the aim to regulate their genes expression in response to increased cell density. In this study, a bacterium (L8A) isolated from dental plaque biofilm was identified as *Citrobacter amalonaticus* by matrix-assisted laser desorption/ionization time-of-flight (MALDI-TOF) mass spectrometry (MS). Its *N*-acylhomoserine-lactone (AHL) production was screened by using two types of AHL biosensors namely *Chromobacterium violaceum* CV026 and *Escherichia coli* [pSB401]. *Citrobacter amalonaticus* strain L8A was identified and confirmed producing numerous types of AHL namely *N*-butyryl-L-homoserine lactone (C4-HSL), *N*-hexanoyl-L-homoserine lactone (C6-HSL), *N*-octanoyl-L-homoserine lactone (C8-HSL) and *N*-hexadecanoyl-L-homoserine lactone (C16-HSL). We performed the whole genome sequence analysis of this oral isolate where its genome sequence reveals the presence of QS signal synthase gene and our work will pave the ways to study the function of the related QS genes in this bacterium.

In the past few generations, dental scientists and microbiologists as well as microbial ecologists have researched on the identification of oral microbes which have led to oral diseases. Dental plaque has been reported as an important microhabitat for oral micro-organisms^1^. Dental plaque forms naturally on the teeth and aids in colonization of exogenous species, which disturbs the microbial homeostasis within oral cavity and becomes predisposed sites to the disease^2^. This diseased dental plaque influences the changes in environmental conditions and plays a vital role in the site specific diseases such as dental caries, gingivitis and periodontitis^3^. In addition, dental plaque which acts as a reservoir of Gram-negative bacteria and periodontium as a reservoir of inflammatory mediators indirectly play a role in systemic diseases such as bacteremia, cardio-vascular diseases, bacterial pneumonia, diabetes mellitus and low birth weight^4^. Previous researches show that a minimum number of 800 bacterial species are present in the oral cavity^5^,^6^ out of which total estimate of 415 species and 68 unseen species were reported from sub-gingival dental plaque^5^. The number of oral bacteria may be increasing with the advancement of biotyping techniques, for example mass sequencing technologies^7^,^6^, mass spectrometry analysis such as MALDI-TOF^8^ and next generation sequencing^9^.

On the other hand, quorum sensing (QS) has been well studied as bacteria genes regulation by signalling molecules termed autoinducers which will increase according to the cell density^10^,^11^. *N*-acylated-L-homoserine lactones (AHLs) and autoinducer AI-2 are the two common classes of autoinducers used by Gram-negative bacteria in their QS system^12^. With the availability of a numbers of AHL biosensors, this has greatly facilitated the screening of AHL production by using the *lux, gfp* or *lacZ* AHL biosensors reporter gene fusions or pigment induction^13^,^14^. AHL varies in acyl group length (C4 to C18), presence or absence of carbon-carbon double bond in the fatty acid chain and substitution of C3 (hydrogen, oxo- and hydroxyl group). To date, QS is an attractive approach for antimicrobial therapy in the treatment of infectious disease^15^ as it does not involve the use of antibiotics.

*Citrobacter amalonaticus* (formerly known as *Levinea amalonaticus*) is a Gram-negative rod-shaped facultative anaerobe with chemoheterotrophic metabolism^16^. The genus *Citrobacter* is described as an opportunistic human pathogen as it has the potential to cause neonatal meningitis^17^ and urinary tract infections^18^. It can be found in urine, sputum, soft tissue exudates and even water or food^17^,^19^. However, there is no report reveals the oral microbe associated *Citrobacter* species and the valuable information about the genomic study on *Citrobacter amalonaticus* is therefore, limited. To-date, next generation sequencing technology has revolutionized the genomic research by generating sequences data in hours or days. The aim of this study is to understand the occurrence of QS in *C. amalonaticus* isolated from dental plaque by studying the AHL production profile and insights gained from the annotated genomic features of *C. amalonaticus* strain L8A.

## Results

### Strain L8A identification

**Figure 1:**
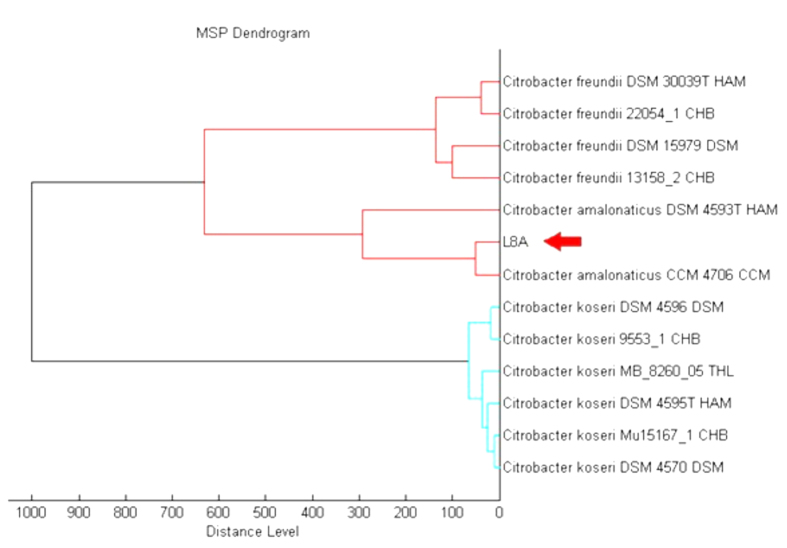
Score-orientated dendrogram of strain L8A. Bacterial strain L8A was clustered hierarchically based on the protein mass spectra patterns with distance value normalized to maximum value of 1,000.

Initial microbial identification of strain L8A was done by Microflex MALDI-TOF Mass Spectrometry system. MALDI-TOF MS has been regarded as a reliable tool for microorganism identification^20^. A number of researchers reported the feasibility of MALDI-TOF MS analysis was able to classify and differentiate clinical isolates to species level^21^,^22^. Using MALDI-TOF mass spectrometry, strain L8A was identified at species level with score value of 2.390 and it was best matched to *Citrobacter amalonaticus*. The MALDI Biotyper MSP software was used as further support to generate the dendrogram of *C. amalonaticus* L8A with its closest match (Fig. 1).

### QS signaling molecules production in *C. amalonaticus* L8A

**Figure 2:**
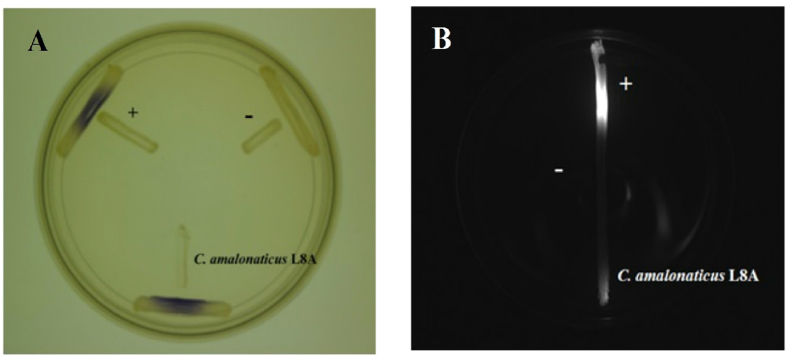
Preliminary AHL screening with cross streak bioassay. (**A**) Short chain AHL screening of strain L8A with CV026. *E. carotovora* PNP22 (negative control “−”) and *E. carotovora* GS101 (positive control “+”) were included for comparison. (**B**) AHL screening of strain L8A with *E. coli* [pSB401]. *E. carotovora* PNP22 (negative control “−”) and *E. carotovora* GS101 (positive control “+”) were included for comparison.

Production of QS activity by strain L8A was detected when it was cross-streaked with *C. violaceum* CV026 and *E. coli* [pSB401] AHL biosensors. Both purple violacein pigmentation (Fig. 2A) and bioluminescence activities (Fig. 2B) were induced by strain L8A at the meeting point between two strains. In particular, L8A showed positive result with both bioreporters used in this study.

**Figure 3:**
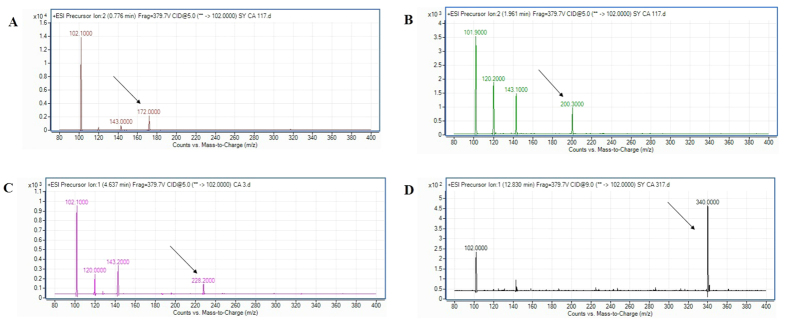
Mass spectrometry analysis of spent supernatants extract *C. amalonaticus* strain L8A. (**A**) Mass spectra of C4-HSL (*m/z* 172.0000) (marked by arrow). (**B**) Mass spectra of C6-HSL (*m/z* 200.3000) (marked by arrow). (**C**) Mass spectra of C8-HSL (*m/z* 228.2000) (marked by arrow). (**D**) Mass spectra of C16-HSL (*m/z* 340.0000) (marked by arrow).

The extracted AHL from spent culture supernatant of strain L8A was analyzed using the high resolution triple quadrupole LC/MS mass spectrometry (MS) system and confirmed that *C. amalonaticus* L8A produced four different types of AHL molecules; namely *N*-butyryl-L-homoserine lactone (C4-HSL) (Fig. 3A), *N*-hexanoyl-L-homoserine lactone (C6-HSL) (Fig. 3B), *N*-octanoyl-L-homoserine lactone (C8-HSL) (Fig. 3C) and *N*-hexadecanoyl-L-homoserine lactone (C16 HSL) (Fig. 3D).

### Data analysis on genomic features of strain L8A

**Figure 4:**
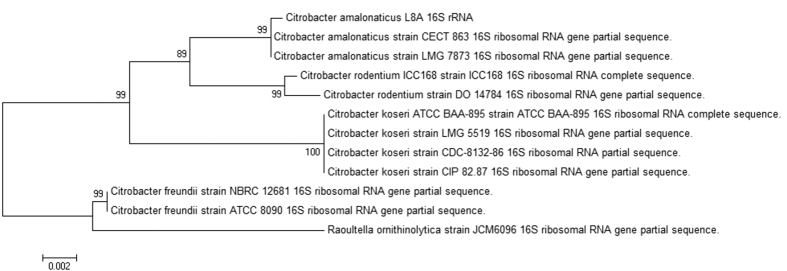
Maximum likelihood phylogenetic tree of *C. amalonaticus* L8A 16S rRNA sequence and other closely related sequences. The phylogenetic analysis of 16S rRNA of the strain L8A was hierarchical clustered under *C. amalonaticus* using MEGA 5.1.

*De novo* genome assembly of *C. amalonaticus* L8A using CLC Genomics Workbench generated 110 contigs with the genome size of 5,273,145 bp and average 40-fold coverage. This genome has average GC content of 54.4% and encodes 4972 putative DNA-coding sequences. With RNAmmer^23^ and tRNAscan-SE^24^ tool, 16S, 23S and 5S rRNA genes and 70 tRNA genes were predicted on the chromosome of *C. amalonaticus* L8A. The 16S rRNA gene was found in contig 18 of the genome with the length of 1,542 bp. The 16S rRNA maximum likelihood phylogenetic tree was rooted using *Raoultella ornithinolytica* JCM6096 as outgroup species. Phylogenetic relationship inferred the highest 16S rRNA gene sequence 99% identical to *Citrobacter amalonaticus* (Fig. 4).

**Figure 5:**
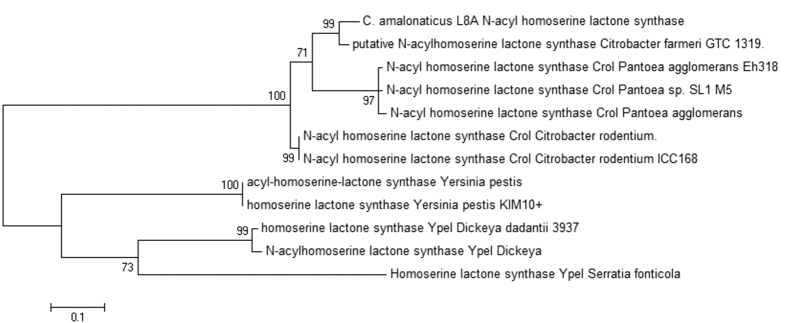
Phylogenetic tree based on QS synthase gene in L8A. *In silico* study of QS synthase gene of genome strain L8A was determined and represented in maximum likelihood phylogenetic tree.

**Figure 6:**
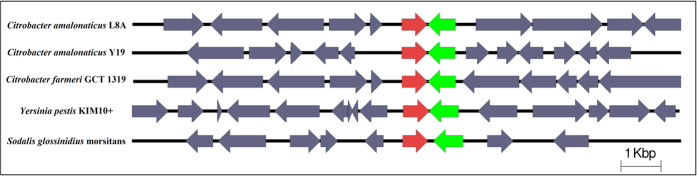
QS synthase gene and regulatory gene in L8A. *C. amalonaticus* L8A QS genes were synteny analyzed using Easyfig (red arrow indicated the QS synthase gene and green arrow indicated the QS transcriptional regulator gene).

AHL-mediated QS system consists of three fundamental components namely *LuxI*-type autoinducer synthase (signal generator), AHL ligand (the signal itself) and LuxR-type receptor (cognate receptor)^25^,^26^,^12^. LuxI-type autoinducer synthase gene was determined and extracted from the genome sequence of *C. amalonaticus* L8A for further phylogenetic study. The 212 bp of putative protein-coding gene of *C. amalonaticus* L8A *N*-acylhomoserine lactone synthase was grouped with *C. farmeri* GTC1319 QS synthase gene (Fig. 5). *C. amalonaticus* L8A QS gene was further annotated using RAST server. The genome feature of QS homoserine lactone synthase gene and QS transcriptional activator gene were found in contig 12. The synteny analysis of annotated QS features were illustrated in Fig. 6 using Easyfig^27^ version 2.2.

**Figure 7:**
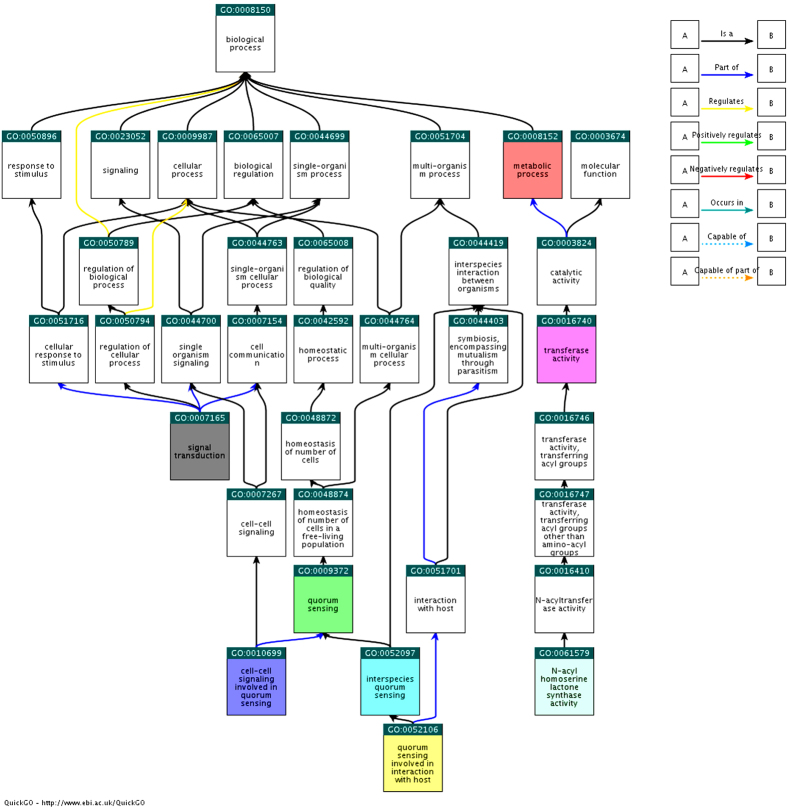
Comparison chart of eight QS-associated Gene Ontology terms using QuickGO (showing in highlight). Different relationships between the terms are indicated by unique colour of arrows as depicted in the right panel.

Association between these AHL synthase related terms and gene products is given in Fig. 7. As displayed in the comparison chart, interspecies QS and QS involved in interaction with host as well as implication of cell-cell signaling were predicted to happen in subclasses of QS which was classified as a type of homeostasis of number of cells within a population of free-living cells such as oral microbiome under biological process. Symbiotic interaction inclusive of interaction with host could be encompassed by genome L8A in oral cavity through mutualism or parasitism. In molecular function, mechanism of *N*-acylhomoserine lactone synthase activity in L8A was simulated and found to be associated with transferase activity. Occurrence of this transferase activity in genome L8A involved in catalysis of transferring acyl group to nitrogen atom in AHL. The term ‘*N*-acylhomoserine lactone synthase activity’ was linked to QS pathway via metabolic process under biological process.

### Multiple genomes comparison

**Figure 8:**
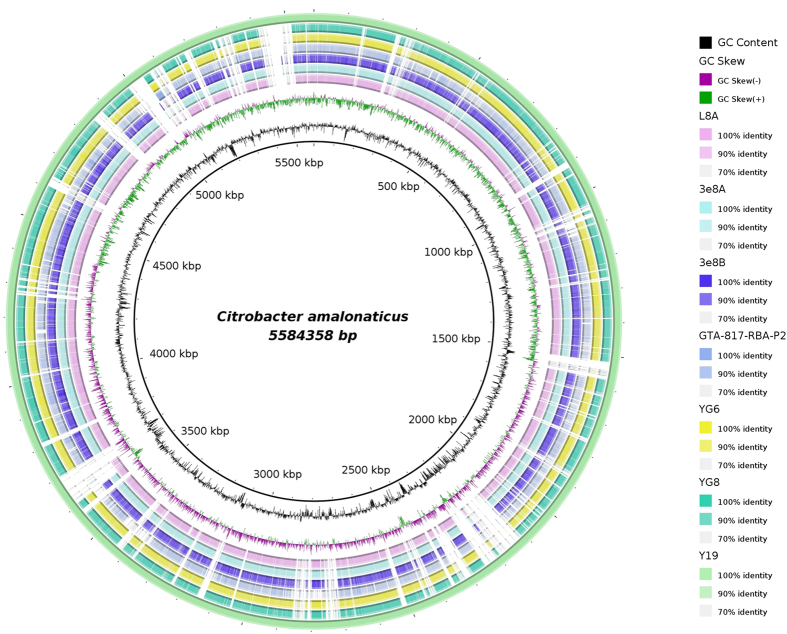
BRIG visualization of multiple *C. amalonaticus* genomes available from NCBI database. The innermost rings show GC skew (purple/green) and GC content (black). The third innermost ring shows L8A genome and followed by strains 3e8A (CDQV01) and 3e8B (CDQX01) isolated from undernourished Malawian children’s gut. The remaining rings represent the ground beef isolate GTA-817-RBA-P2 (LAMY01) and environmental strains YG6 (LIGA01) and YG8 (LIGB01) prior the outermost ring with complete genome strain Y19.

To date, there are only seven whole genome sequences publicly available from NCBI database inclusive L8A genome. Genome comparison of these seven *C. amalonaticus* genomes with clinical isolates (n = 3), environmental (n = 2), food (n = 1) and industrial (n = 1) was performed using BRIG (Fig. 8) in circular visualization. Among these seven genomes, six were draft genomes and one was complete genome which was used as reference genome. Average genome size was 5.139 Mb with GC content of 53.35%.

### Nucleotide sequence accession number

This Whole Genome Shotgun project has been deposited at DDBJ/EMBL/GenBank under the accession JMQQ00000000. The version described in this paper is version JMQQ01000000.

## Discussion

*Citrobacter* sp. is an opportunistic pathogen that infrequent infects neonates and in immunocompromised adults or older children^28^ who constantly follow the prophylactic β-lactam therapy^29^. However, *C. amalonaticus* infection is rare and only few cases have been described worldwide. In 2005, a 53 years old American male tourist was admitted to hospital and diagnosed with enteric fever syndrome caused by *C. amalonaticus* infection. He had initial cefriaxone treatment followed by co-trimoxazole for a period of 23 days^30^. Other case reports responded to bacterial infection with this species mostly happened in young pregnant female with underlying conditions such as urinary tract infection, vesico vaginal fistula and acute pyelonephritis^31^,^32^,^33^.

Bacteria in *Citrobacter* genus are currently divided into 11 species^19^. They are genotypically and biochemically diverse. In order to decrease the possibility of misidentification, molecular method and whole genome sequencing of strain L8A was performed. Both MALDI-TOF mass spectrometry and 16S rRNA gene results confirmed strain L8A is *C. amalonaticus*. Compared to the Human Oral Microbiome Database (HOMD)^34^ as part of Human Microbiome Project (HMP) and other literatures, surprisingly this strain has not been reported to be associated with oral cavity. When the 16S rRNA gene of *C. amalonaticus* L8A was searched (BLASTn) against the HOMD 16S rRNA reference sequences, the results returned the highest percentage identical to *Enterobacter hormaechei* references is only 97.7%, followed by *Kluyvera ascorbata* (97.5%) and *E. coli* (97.4%). This finding disclosed the existence of *C. amalonaticus* in the human oral cavity.

In general, Gram-negative bacteria communicate via expression of exogenous small diffusible molecules known as *N*-acyl homoserine lactone (AHL) to mediate gene expression in a cell density dependent manner. Frias and colleagues reported that QS in *Porphyromonas gingivalis* play a role in controlling the production of its virulence factor in oral cavity^35^. Besides, previous research suggested that AHL gene in *Citrobacter rodentium* has an unexpected role in affecting the mouse’s virulence factors in the manner of attaching and effacing lesions through QS^36^.

Typically, LuxI-like autoinducer synthase and LuxR-like receptor are clustered in pair. There are some articles reported that functional LuxI/LuxR pairs are not located on common bacterial chromosome or plasmids^37^,^38^ or absence of LuxI autoinducer synthase in bacterial QS circuit^39^. In order to identify the QS signal synthase and its cognate receptor genes, we performed the whole genome sequencing on *C. amalonaticus* L8A. Upon analysis of its genome sequence, we found that LuxI/LuxR functional pair of *C. amalonaticus* L8A is genetically adjacent and clustered on the same chromosome. The detected QS activity in this strain could be an alternative solution for treatment as most Gram-negative bacteria depend on QS to regulate virulence factors expression^40^.

Additionally, the pathway analyses of Gene Ontology terms had conveyed a simplified biological insight for large proteomic and genomic datasets. Multiple GO terms could be used for a single gene annotation manually or electronically with references. The QS pathway showing that interspecies QS and QS interplay with host occurred in L8A genome. Interspecies signaling between bacterial species via small diffusible AHL molecules has been suggested by Eberl and Tümmler^41^; it happened in patients with cystic fibrosis between *Pseudomonas aeruginosa* and *Burkholderia cepacia*. QS involves in interaction with host, in particular, a process applied by community of single-cell L8A microorganism living in intimate contact with host and controlling the population density with the concentration of signaling molecules. The host here referred to a larger organism which responded to symbiotic interaction^42^,^43^. In this case, strain L8A is able to use their available proteins to adhere to each other and form layer of biofilm. However, the information about the possible role of AHL-regulated QS of *C. amalonaticus* is still a mystery. Therefore, it is vital to link the possible proteins to signaling pathway for later experiment design and evaluation and future work in finding out anti-QS-based solution to control the oral cavity diseases.

In addition to this, overview of sequence feature information is able to be visualized in the context of sequence analysis results generated through the comparative genomics tool. The sole complete genome *Citrobacter amalonaticus* Y19 (CP011132) was assigned as reference genome in this comparative study. This strain was reported with capability to produce hydrogen from oxidizing toxic carbon monoxide^44^. The output image shows differences and similarity between the query genomes and reference genome, where BLAST matches are indicated as coloured sliding scale at side. From the comparison study, we can predict the possible antimicrobial resistance genes, important genomic traits for industrial application and other genomic features.

In summary, for the first time, we have profiled the unusual various types of AHLs produced by *C. amalonaticus* strain L8A isolated from dental plaque. Coupling with next generation sequencing technology and systematic bioinformatics analysis, our study has provided insights into *C. amalonaticus* genome features and invaluable information about this bacterial strain notably on the QS system. Besides this, the genome *C. amalonaticus* L8A reported in this work can be exploited as reference genome for future comparative, functional and clinical study of *Citrobacter* genus. The genome sequence may be useful for research on any related genes through its nucleotide sequences analysis, orientation, upstream and downstream regulatory elements and gene products, as well as its miscellaneous genomics features. By elucidating the QS genes in *C. amalonaticus*, this will pave the way to understand the QS regulation in this bacterium. In addition, this work also illustrated that oral cavity that could be potential reservoir for QS pathogen and hence more intense research should be carried out.

This is the first documentation reported *C. amalonaticus* possesses QS activity, whole genome sequencing, and its AHL production namely C4-HSL, C6-HSL, C8-HSL, and C16-HSL.

## Materials and Methods

### Ethics statement

All of the procedures were carried out in accordance with guidelines approved by the Medical Ethics Committee Faculty of Dentistry, University of Malaya (DFRD-1302/0033-L). Informed consent was obtained from all subjects prior to commencement of this study.

### Sample Collection and Bacterial Strains Isolation

Dental plaque biofilms samples were collected from healthy patients who were scheduled to have their teeth extracted due to deep caries at the Primary Care Unit, Faculty of Dentistry. Soft and loose dental plaque around the buccal and proximal surfaces of the carious teeth was gently curated using sterile Gracey curettes. The collected samples were placed into a sterile microcentrifuge tube with PBS buffer concentration of 0.01 M at pH 7.4, maintained at 4 °C and transported to the laboratory for further analysis within 24 hours. Briefly, saline sample was vortex-homogenized for 1 min prior to 100 μL in aliquots spread on Lysogeny Broth (LB) medium and aerobically cultured overnight at 37 °C. The isolated bacterial strains from dental plaque samples were repeatedly streaked on LB agar to obtain the pure colony.

### MALDI-TOF application for strain identification

MALDI-TOF mass spectrometry (MS) was used to identify the bacterial strains. Briefly, pure colony of interested strain was smeared thinly onto each well of MSP 96 MALDI Target plate prior to adding 1μl of HCCA MALDI matrix and allow the mixture to co-crystallize^45^. The target plate was then subjected to automated MALDI Biotyper bench-top mass spectrometer using FlexControl software version 3.3. Bruker MALDI Biotyper Real Time Classification (RTC) (Version 3.1) software was implemented to analyze the generated spectra by standard pattern matching with default settings and identification was provided with score value^46^. In addition, the score-oriented dendrogram was constructed using MALDI Biotyper MSP creation method.

### Preliminary screening of AHLs

Two different short chain AHLs bacterial biosensors namely *Chromobacterium violaceum* CV026 and *Escherichia coli* [pSB401] were employed for the preliminary AHLs screening of bacterial isolates. *Erwinia carotovora* GS101 and *E. carotovora* PNP22 were used as positive and negative controls, respectively^45^,^47^. These AHLs biosensors and controls were cultivated overnight in LB medium at 28 °C. Bacterial isolates were then screened for its AHLs production by cross-streaking with AHL biosensors. Purple violacein pigmentation formed in *C. violaceum* CV026 and intensity of bioluminescence of *E. coli* [pSB401] will increase if exogenous short chain AHL was detected^48^,^49^. The bioluminescence image was electronically documented by Hamamatsu Photonics photon camera (Hamamatsu, Japan).

### Extraction of AHLs

The QS positive strain was grown overnight in LB broth buffered with 50 mM 3-(*N*-morpholino) propanesulfonic acid (MOPS) (pH 5.5) in 37 °C incubator shaker with 200 rpm. The acidified organic solvent (0.1% v/v glacial acetic acid) ethyl acetate was used to extract the supernatant culture twice as reported previously^50^. The extracted supernatant was evaporated to complete dryness in fume hood. The dried extract was resuspended in 200 μL of acetonitrile (HPLC grade) and vortex vigorously to dissolve the extracted AHLs. The mixture was then centrifuged and any insoluble residue was discarded. The dissolved sample (100 μL) was inserted into sample vials for mass spectrometry analysis.

### AHL Profiling by High Resolution Tandem Liquid Chromatography Mass Spectrometry (MS)

The extracted AHLs profile of the bacterial strain was analyzed using high resolution MS namely Agilent 1290 Infinity LC system and Agilent 6490 Triple Quadrupole LC/MS system (Agilent Technologies Inc., Santa Clara, CA, USA) associated with Agilent ZORBAX Rapid Resolution High Definition SB-C18 Threaded Column. Mobile phases A and B used in this study were water and acetonitrile (both mobile phases added with 0.1% v/v formic acid), respectively with an initial ratio of 80:20. The parameters included flow rate, temperature were set as described previously^46^,^51^. The precursor ion scan mode will scan through m/z value from 150 to 400 and target the *m/z* 102 [M+H]^+^ product ion which is the characteristic of the lactone ring moiety and indicates the presence of AHL. The generated EI mass spectra were then compared to the standard AHL profile based on the retention index using the Agilent MassHunter software.

### Library preparation for next generation sequencing

Total DNA of the strain was extracted using MasterPure^TM^ Complete DNA and RNA Purification Kit (Epicentre Biotechnologies, Madison, WI, USA), performed as recommended in the manufacturer protocol. The quality and quantity of DNA were examined using NanoDrop 2000 spectrophotometer (Thermo Fisher Scientific, USA) and Qubit 2.0 fluorometer (Life Technologies, Carlsbad, CA), respectively, prior to library preparation using Illumina Nextera DNA Sample Preparation Kit (Illumina, USA). The quality control of the DNA library template was analyzed on Agilent 2100 Bioanalyzer system using High Sensitivity DNA Analysis Kit (Agilent Technologies, USA) whereas the concentration was determined using Illumina Eco qPCR machine, as described in the KAPA Library Quantification Kits for Illumina sequencing platforms (KAPA BioSystems, Boston, MA, USA). By calculating from qPCR reading, library template was diluted to 2 nM in Tris-HCl buffer (10mM, pH 8.5 with 0.1% v/v Tween 20). The DNA library was denatured and further diluted to 10 pM before loaded into cartridge for MiSeq (Illumina, USA) next generation sequencing platform, guided by the MiSeq Control Software (MSC) interface.

### Genome assembly and annotation

The raw reads generated by MiSeq were first quality evaluated using FastQC analyzer^52^ prior to trimming the bad reads based on their base quality, filtering according to read length and ambiguous nucleotides were excluded using CLC Genomics Workbench^53^ version 7.0.4. In this genome data analysis, raw reads with average quality value lower than Q20 were excluded together with ambiguous nucleotide. In order to obtain the best assembly result, *de novo* genome assemblies were performed multiple times using different word size parameter from post-filtered reads with minimum read-length of 15 nucleotides.

A rapid prokaryotic genome annotation system (Prokka)^54^ was used to annotate the draft genome of strain L8A. All predicted genes by Prodigal^55^ version 2.60 were searched against the databases of known sequences (NCBI-NT/NR databases) using BLAST tool in order to assign appropriate functional annotation. In addition, the genome of strain L8A was subject to automatic SEED-based annotation using Rapid Annotation Subsystems Technology (RAST) server^56^. Easyfig version 2.2 was implemented for downstream synteny analysis of QS synthase and regulator genes.

### Phylogenetic analysis

The predicted protein coding sequences of 16S rRNA and QS synthase gene sequences were extracted from strain L8A whole genome sequence with the aim to study their phylogenetic relationships with the related gene sequences available in public databases. Molecular Evolutionary Genetic Analysis version 5.1 (MEGA)^57^ tool was used to construct maximum likelihood (ML) phylogenetic trees with GTR nucleotide substitution model and 1,000 bootstrap values.

### *In-silico* discovery QS system

The analysis of Gene Ontology terms or physical interaction network associated with amino acid regulated genes based on their involvement in cellular component, biological process and molecular function. With the accession number of *N*-acyl-homoserine lactone synthase of strain L8A (A0A083ZD14) which provided by UniProt Knowledgebase (UniProtKB), pathway was determined by fast web-based GO browser, QuickGO^58^. Five AHL synthase related GO terms coupled with three child terms from the term of ‘quorum sensing’ were used to construct the comparison chart.

### Comparative genome studies of strain L8A

To perform genomes comparison for strain L8A, the assembled contigs were ordered and re-oriented by aligning them to a closely-related reference genome using Mauve^59^ and concatenated to produce a pseudo-molecule. The comparison files between the query pseudo-molecule genome sequence and reference genome sequence were created using the local BLASTn program to show the percentage of match hit between them. The structural similarities and differences between the genomes sequences were then visualized using BLAST Ring Image Generator (BRIG)^60^ software.

## Additional Information

**How to cite this article**: Goh, S.-Y. *et al*. Quorum sensing activity of *Citrobacter amalonaticus* L8A, a bacterium isolated from dental plaque. *Sci. Rep.* **6**, 20702; doi: 10.1038/srep20702 (2016).
